# Editorial: The digitalization of neurology—volume II

**DOI:** 10.3389/fdgth.2026.1806851

**Published:** 2026-03-04

**Authors:** Daniel B. Hier, Michael D. Carrithers, Jorge M. Rodríguez-Fernández, Benjamin Kummer

**Affiliations:** 1 Department of Neurology and Rehabilitation, University of Illinois at Chicago, Chicago, IL, United States; 2 Kummer Institute for Artificial Intelligence and Autonomous Systems, Missouri University of Science and Technology, Rolla, MO, United States; 3 Department of Neurology, University of Texas Medical Branch, Galveston, TX, United States; 4 Department of Neurology, Icahn School of Medicine at Mount Sinai, New York, NY, United States

**Keywords:** ALS, board examination, digitalization, large langagage model, neurology, optic disc, step count

Our 2023 editorial, *Digitalization of neurology*, highlighted how neurology was being reshaped by the steady accumulation of digital signals—including telemedicine encounters, electronic health records, neuroimaging, electrophysiology, remote monitoring technologies, and wearable and mobile sensors—enabling more scalable phenotyping and continuous, real-world measurement. We also noted that the documentation burden imposed by electronic health records remained largely unaddressed in 2023.

Volume II arrives as this trajectory takes a sharp turn. The emergence of large language models (LLMs) and, more broadly, foundation models (including vision—language models) is transforming not only what data we collect and what models we build, but also how outputs are interpreted and integrated into clinical reasoning. As these systems enter practice, they are beginning to reshape neurological workflows in the Research Topic, synthesis, and summarization of clinical information, and may finally offer a realistic path to reducing documentation burden.

More fundamentally, digitalization in neurology is evolving from the digitization of inputs for neurologic reasoning—clinical notes, neurophysiologic signals, radiologic images—to the use of AI systems that actively participate in that reasoning. Foundation models increasingly function as interpreters, translating the complex, often opaque outputs of specialized predictive algorithms into clinically meaningful neurological insights.

**Figure 1:**
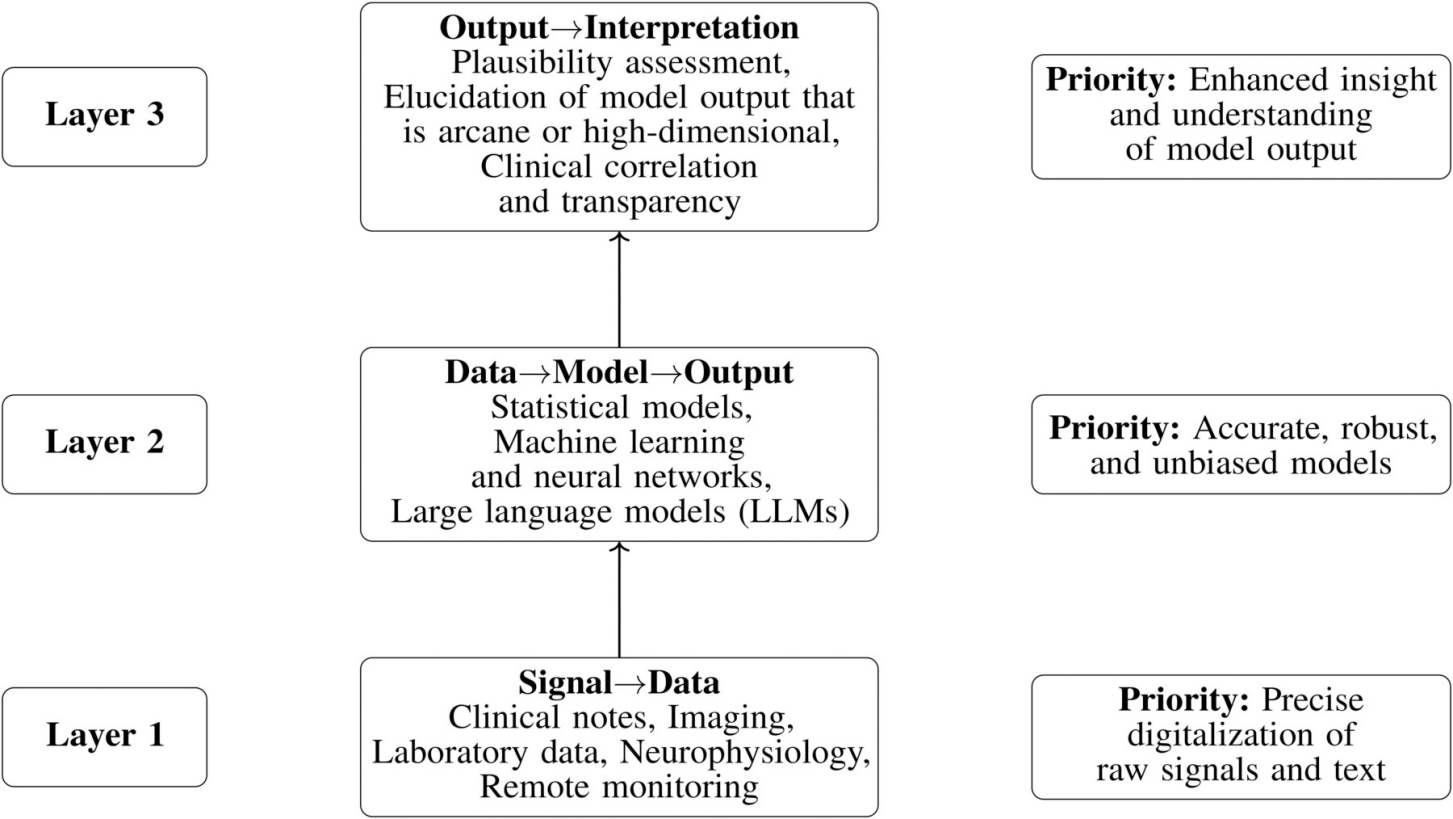
A three-layer framework for AI-enabled neurology, emphasizing the dependencies between data quality, model capability, and interpretation. Progress depends on coordinated advances across all three layers. We suggest that large language models will play a disproportionate role at Layer 3, where complex and high-dimensional model outputs require careful interpretation, contextualization, and clinical validation. This figure was created within Overleaf.

The four accepted articles in this second volume reflect this shift from enhanced signal digitalization toward more capable AI models with expanded interpretive roles (Figure 1). Three focus on LLMs or vision—language models—examining how they perform, how they fail, and how prompt design shapes clinically adjacent outputs—while the fourth returns to a foundational theme of digital neurology: remote, continuous measurement as a candidate biomarker in neurodegenerative disease. Together, these contributions illustrate a field moving from isolated digitalization (signal→data) toward an ecosystem that links digital measurement, predictive modeling, and digital intelligence (signal→data→model→output→interpretation).

*Large language models for neurology: a mini review* spans all three levels of our framework—better data, better models, and better interpretations—by surveying how LLMs can augment neurological reasoning, workflow, documentation, and multimodal data use while remaining constrained by bias, uneven reliability, and regulatory uncertainty. Wunsch and Hier synthesize evidence that advanced models now approach or exceed human performance on neurology board-style questions and selected tasks such as lesion localization and seizure focus identification, yet often underperform neurologists on complex, real-world diagnostic cases unless heavily specialized or fine-tuned. They highlight expanded roles for LLMs in neurologist workflow, including note acquisition through ambient AI, expedited patient education, EHR summarization, prior authorization, and disease coding. They also review disease-specific applications of LLMs to stroke, dementia, Parkinson’s disease, and multiple sclerosis. Looking ahead, the review envisions neurology-focused, ontology-aware, multimodal models that fuse clinical text, neuroimaging, electrophysiology, and multi-omics into digital-twin simulations and interoperate natively with FHIR and standard vocabularies and ontologies, but emphasizes that realizing this vision will require robust governance—bias mitigation, privacy protection, appropriate device regulation, retrieval-augmented architectures to stay current, and close alignment with the priorities of neurologists so that emerging systems reduce documentation burden without eroding expert judgment.

*Evaluation of multiple generative large language models on neurology board-style questions* fits within Layer 2 (better models) of our framework and highlights the growing ability of modern LLMs to serve as repositories of neurological knowledge and limited neurology board-style reasoning. Almomani et al. benchmarked eight models against neurology residents on 107 text-only board-style questions across seven subspecialties and Bloom-stratified cognitive levels. ChatGPT-5 and ChatGPT-4o led overall and outperformed residents, while Gemini 2.5 was close behind and also exceeded residents, matching top models on higher-order items; earlier models (e.g., Gemini v1/Bard, Claude, ChatGPT-3.5) performed at or below resident level with domain variability. However, calibration was poor: self-reported confidence did not reliably track answer correctness (confidence—accuracy correlations were weak and sometimes negative), and agreement with expert Bloom labels was low (κ in the “slight” range). These findings support using LLMs as supervised educational adjuncts rather than substitutes for examinations or unsupervised clinical use.

*Performance of vision-language models for optic disc swelling identification on fundus photographs* also fits within Layer 2 (better models) of our framework (Figure 1) and serves as a cautionary counterpoint to LLM enthusiasm. Li and colleagues evaluate whether general-purpose vision–language models can distinguish swollen from normal optic discs on fundus photographs and show that, although several models perform better than chance, none approach the very high accuracies of task-specific convolutional neural networks; general-purpose VLMs achieve at best a Youden index of 0.23 and accuracy of 70.5%, whereas prior CNN systems for the same task routinely exceed 90% accuracy. Increasing prompt complexity or applying general medical fine-tuning does not consistently improve performance, underscoring that the added flexibility and apparent explainability of VLMs do not compensate for their inferior discrimination. The authors conclude that ophthalmology-specific multimodal models—and rigorous benchmarking—will be needed before such tools can be safely integrated into clinical neuro-ophthalmic decision support, and that mature “older” deep learning pipelines remain the benchmark for safety-critical image classification.

*Exploratory analysis of smartphone-based step counts as a digital biomarker for survival in ALS patients* fits squarely within Layer 1 (better data) of our framework and asks whether passively captured, everyday step counts can serve as a meaningful marker of disease progression and survival in amyotrophic lateral sclerosis. The study revisits a core promise of digital neurology: continuous, real-world measurement that can detect clinically relevant change missed by intermittent clinic visits, and do so at scale. In the context of volume II, it also anticipates a likely convergence in the coming decade, in which sensor-derived longitudinal phenotypes—such as step counts—are interpreted alongside narrative clinical data (notes, messages, symptom descriptions), with foundation models as potential integration layers. Even without explicitly invoking LLMs, this work strengthens the empirical basis for remote monitoring using digital metrics, both as clinically meaningful endpoints and as inputs to downstream predictive models.

The Research Topic *The digitalization of neurology—volume II* reflects a broader shift in medicine: a progression from digitizing data, to building increasingly sophisticated models, to more sophisticated interpretations of complex model outputs (Figure 1). The near-term agenda should focus on three priorities: better data inputs, better models, and better interpretations. In this sense, volume II is a pivot from volume I. The question is no longer whether neurology will be digital, but how to extract maximal clinical value from increasingly capable AI systems—with large language models playing a central role in model construction and model interpretation.

